# Rheological Behavior and Dynamic Mechanical Properties for Interpretation of Layer Adhesion in FDM 3D Printing

**DOI:** 10.3390/polym14132721

**Published:** 2022-07-03

**Authors:** Supaphorn Thumsorn, Wattanachai Prasong, Takashi Kurose, Akira Ishigami, Yutaka Kobayashi, Hiroshi Ito

**Affiliations:** 1 Research Center for GREEN Materials and Advanced Processing, Yamagata University, 4-3-16 Jonan, Yamagata 992-8510, Japan; kurose.takashi@sist.ac.jp (T.K.); akira.ishigami@yz.yamagata-u.ac.jp (A.I.); kobayashi.y@yz.yamagata-u.ac.jp (Y.K.); 2 Department of Industrial Engineering, Faculty of Engineering, Pathumwan Institute of Technology, 833 Rama I Road, Wangmai, Pathumwan, Bangkok 10330, Thailand; wattanachai@pit.ac.th; 3 Department of Mechanical Engineering, Faculty of Science and Technology, Shizuoka Institute of Science and Technology, 2200-2 Toyosawa, Shizuoka 437-8555, Japan; 4 Graduate School of Organic Materials Science, Yamagata University, 4-3-16 Jonan, Yamagata 992-8510, Japan

**Keywords:** composite, dynamic mechanical analysis, 3D printing, layer adhesion, morphology

## Abstract

Commercial filaments of poly(lactic acid) (PLA) composites with particulate filler, carbon fiber, and copper powder with different contents were fabricated by FDM 3D printing in XZ-direction at bed temperatures of 45 °C and 60 °C. The effects of additives and bed temperatures on layer adhesion, fracture behavior, and mechanical performance of the PLA composites 3D printing were evaluated. Rheological properties informed viscous nature of all filaments and interface bonding in the PLA composites, which improved printability and dimensional stability of the 3D printing. Crystallinity of the PLA composites 3D printing increased with increasing bed temperature resulting in an improvement of storage modulus, tensile, and flexural properties. On the contrary, the ductility of the 3D printing was raised when printed at low bed temperature. Dynamic mechanical properties, the degree of entanglement, the adhesion factor, the effectiveness coefficient, the reinforcing efficiency factor, and the Cole–Cole analysis were used to understand the layer adhesion, and the interfacial interaction of the composites as compared to the compression molded sheets. SEM images revealed good adhesion between the additives and the PLA matrix. However, the additives induced faster solidification and showed larger voids in the 3D printing, which indicated lower layer adhesion as compared to neat PLA. It can be noted that the combination of the additives and the optimized 3D printing conditions would be obtain superior mechanical performance even layer adhesion has been restricted.

## 1. Introduction

Fused deposition modeling (FDM) 3D printing has been widely utilized in various industries due to its custom design and cost effectiveness [1,2,3,4,5,6,7,8]. FDM 3D printing products have formed by the layer-by-layer deposition, which exhibited anisotropic characteristic and drawbacks such as poor interlayer adhesion, incomplete printing, shrinkage and low dimension accuracy resulting in low mechanical performance and their structural failure [3,5,7,8,9]. The selection of filaments and the controlled printing conditions can be optimized for improving the drawbacks and obtaining superior mechanical performance of the FDM 3D printing products [1,2,10,11,12]. Polymer filaments are feedstocks for the FDM 3D printing. Acrylonitrile butadiene styrene (ABS), poly(lactic acid) (PLA), and poly(ethylene terephthalate glycol) (PETG) are commonly filaments for the FDM 3D printing, which have good printability at low printing temperature ranges compared to high performance plastics, such as polycarbonate (PC), polyamide (PA, nylon), polyetherimide (PEI), and poly(ether-ether-ketone) (PEEK) [2,13,14,15,16]. The polymer filaments have been developed by the incorporation of additives, blending and estimated suitable printing conditions to diminish the FDM 3D printing drawbacks [1,5,6,7,9,10,11,12,13,14,15,17,18,19,20,21]. The following research performed guidelines to overcome the drawbacks. The effects of nozzles temperatures, bed temperatures, and annealing conditions on properties of the FDM 3D printing of PLA were reported by Benwood et al. [5]. The increasing in the nozzle and the bed temperatures significantly improved mechanical properties of the PLA 3D printings, which exhibited less voids and have good layer adhesion. Nguyen et al. applied carbon fiber (CF) in the lignin based ABS composite 3D printing, which the CF acted as a bridging between the printed layers and improved the interlayer adhesion in the lignin/ABS-rubber composites [10]. Ferreira et al. [20] noticed that CF highly oriented during the deposition in the 3D printing, which strongly improved tensile modulus but reduced elongation of the CF reinforced PLA composites 3D printing. Additionally, the higher printing temperature resulted in the present of bubble-like structure in the specimens and introduced the nozzle temperature lower than 220 °C for printed the PLA/CF 3D printing. 

Generally, mechanical performance of the FDM 3D printing depends on the layer adhesion in the products. Nevertheless, mechanical properties of composite materials in the FDM 3D printing were improved but limited in the layer adhesion [1,16,22]. To clarify the layer adhesion and interfacial adhesion of the composites 3D printing, rheological studied and dynamic mechanical analysis are used to understand the interfacial adhesion, the interlayer adhesion and properties of polymer composites and the FDM 3D printing [3,4,10,19,23,24,25,26,27,28,29]. Aw, et al. [19] studied the effect of printing parameters on tensile, dynamic mechanical, and thermoelectrical properties of FDM 3D printed conductive ABS (CABS)/zinc oxide (ZnO) composites. An increment of the storage modulus implied the improvement of stiffness and informed good interfacial adhesion between fillers and the matrix in CABS/ZnO composites FDM 3D printing. While the loss modulus and the damping properties decreased with increasing the infill density [19]. Thermal properties and dynamic mechanical properties of PLA/mica composites were reported by Kim, et al. [27]. Mica was the initial nuclei for PLA crystallization and might become the anchored molecules to enhance the interfacial adhesion through mechanical interlocking. Kunjappan, et al. [28] discussed on the reinforcement efficiency factor, degree of entanglement, and the effectiveness of multiwall carbon nanotube (MWCNT) to inform the interfacial adhesion and the effectiveness of MWCNT on properties of poly(trimethylene terephthalate)/polyethylene blend composites. Jyoti, et al. [26] applied the dynamic mechanical analysis to clarify the effectiveness of the MWCNT to reinforce MWCNT/ABS composites. Hence, the rheological behavior, the dynamic mechanical properties, the degree of entanglement density, the adhesion factor and the Cole–Cole analysis are carried out to clarify the flow behavior, the phase structure, the interfacial adhesion and the layer adhesion in the FDM 3D printing of polymer composites. This information would be a benchmark to development polymer composites and printing conditions for superior properties of the polymer composites FDM 3D printing. 

In this research, the roles of additives and the printing bed temperatures on flow behavior, the layer adhesion, thermal properties, mechanical performance, and failure behavior in PLA composites FDM 3D printing were investigated. The phase structure of the PLA composites was analyzed by the Cole–Cole analysis. Dynamic mechanical properties were discussed in term of molecular entanglement, adhesion factor, effectiveness coefficient and reinforcement efficiency factor to interpretation the layer adhesion, the interfacial adhesion, and elucidate mechanical performance and failure characteristic of the PLA composites FDM 3D printing. 

## 2. Materials and Methods

### 2.1. Filaments and Sample Preparation

Four types of commercially available poly(lactic acid) (PLA) filaments including neat PLA and PLA composites with particulate filler, carbon fiber, and copper particle were used as received. The formulations and the processing of the filaments are owned by the manufacturer. Table 1 tabulated compositions and designations of the PLA composites filaments in this study. Since the formulations and the processing of the filaments are owned by the manufacturer, the characteristic of the additives in the filaments was observed by scanning electron microscope (SEM) and solid residual of additives and thermal stability were measured by thermogravimetric analysis (TGA). The contents of particulate filler (P), carbon fiber (CF) and copper particle (Cu) were characterized by thermogravimetric analyzer, which were about 5 wt.%, 14 wt.% and 66 wt.%, respectively, as presented in Table 1. 

**Figure 1:**
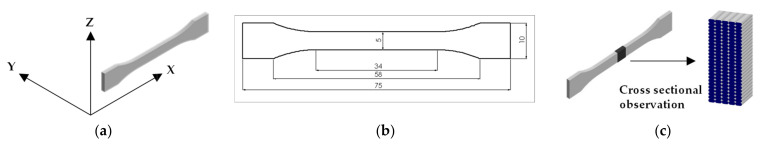
3D printing sample preparation: (**a**) Printing direction; (**b**) size of dumbbell specimen; and (**c**) cross sectional for morphology observation.

The filaments were printed by the FDM 3D printing (da Vinci 1.0 Pro, XYZprinting, Inc., New Taipei City, Taiwan) to bar samples (60 mm long, 10 mm wide, and 2 mm thick) and dumbbell samples (75 mm long, 5 mm wide, and 2 mm thick) in XZ-directions. The nozzle was 0.4 mm-diameter and set at temperature of 210 °C. The printed bed temperatures were varied at 45 °C and 60 °C. Figure 1 depicts the printing direction, the size of the dumbbell sample and the cross sectional for morphology observation. The conditions for 3D printing are summarized in Table 2 [5,11,22]. 

**Figure 2:**
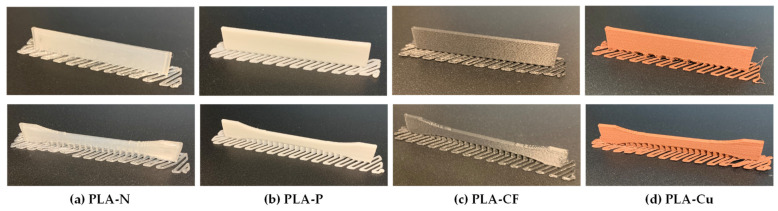
Bar (top) and dumbbell (bottom) 3D printed samples from bed temperature 60 °C: (**a**) PLA-N; (**b**) PLA-P; (**c**) PLA-CF; and (**d**) PLA-Cu.

Figure 2 shows photographs of bar and dumbbell 3D printed samples of PLA composites printed at bed 60 °C. 

In addition, the filaments were cut and compression-molded to 2 mm-thick sheets by a compression molding machine (Mini Test Press MP-WNH, Toyo Seiki Seisaku-sho, Ltd., Tokyo, Japan) at temperature of 200 °C with pressure 10 MPa for 5 min. 

The samples designations such as PLA-N filament, compression molded sheet, 3D printed samples at bed 45 °C and at bed 60 °C are referred as PLA-N-F, PLA-N-C, PLA-N-B45 and PLA-N-B60, respectively. 

### 2.2. Characterization

#### 2.2.1. Morphology

Characteristic of the additives used in the filament was observed from the compression molded sheet of the filaments by SEM (JSM-6510, JEOL Ltd., Tokyo, Japan). Cross sectional morphology of the 3D printed samples as shown in Figure 1c was observed from cryogenic fractured surfaces and tensile fractured surfaces by an optical microscope (VHX950F, Keyence Corporation, Osaka, Japan) and SEM (TM3030plus, Hitachi High-Technologies Corporation, Tokyo, Japan). The surfaces of SEM samples were coated with platinum and observed at an accelerated voltage of 5 kV.

#### 2.2.2. Rheological Properties

Rheological properties were tested with the compression molded sheets of the filaments. Samples were conducted by a rotary rheometer (Modular Compact Rheometer, MCR 302, Anton Paar GmbH, Graz, Austria) using a 25 mm parallel plate. The temperature was set at 210 °C. The oscillatory mode was set with frequency ranges from 0.01 to 1000 rad/s with strain rate of 1.0%.

#### 2.2.3. Thermal Properties

Thermal stability and the residual of the additives of the filaments were analyzed by a thermogravimetric analyzer (TGA, Q50, TA Instruments, New Castle, DE, USA). The weight of sample was about 10 mg. The temperature was set from the ambient to 600 °C at the heating rate of 10 °C/min under nitrogen atmosphere. 

Thermal properties and crystallization behavior of the filaments, the compression molded sheets and the 3D printing samples were characterized using a differential scanning calorimeter (DSC, Q200, TA Instruments, New Castle, DE, USA) under nitrogen atmosphere. The 3D printing samples were cut from the center of dumbbell specimens. Samples about 5 mg were sealed in aluminum pan. The DSC analysis was run on heat-cool-heat cycles set from −70 °C to 200 °C at the heating and cooling rates of 10 °C/min. The cycle was held isothermally 5 min before running the cycle. Crystallinity of PLA in the samples was calculated as described in the literature [5,22] by the following equation.

(1) $$X_{c}\left(\%\right)=\frac{\left(\Delta H_{m}-\Delta H_{cc}\right)}{\Delta H_{f}^{0}\times W_{PLA}}\times 100$$

where $X_{c}$ is the percentage of crystallinity, $\Delta H_{m}$ is the enthalpy of melting, $\Delta H_{cc}$ is the enthalpy of cold crystallization, $\Delta H_{f}^{0}$ is 93.7 J/g for the enthalpy of fusion of fully crystalline PLA [5], and $W_{PLA}$ is the weight fraction of PLA in the filaments.

#### 2.2.4. Dynamic Mechanical Properties

Dynamic mechanical analysis was carried out by the RSA G2 solids analyzer (TA Instruments, New Castle, DE, USA). The bar specimens (30 mm long, 6 mm wide, and 2 mm thick) from the compression molded sheets and the 3D printings were run at 3-point bending mode. The condition was set from room temperature to 120 °C at the heating rate of 3 °C/min and frequency of 1 Hz. Storage modulus (E’), loss modulus (E”) and Tan δ were recorded. 

#### 2.2.5. Mechanical Properties

Flexural and tensile properties were performed using a universal testing machine (Strograph VGS1-E, Toyo Seiki Seisaku-sho, Ltd., Tokyo, Japan). Flexural testing was done according to ISO 178 with bar specimens (*n* = 3) at span length of 32 mm and testing speed of 2 mm/min. Tensile testing was carried out according to ISO 527-2 type 1 BA with dumbbell specimens (*n* = 5) at gauge length of 30 mm and testing speed of 10 mm/min. 

## 3. Results and Discussion

### 3.1. Morphology of the Compression Molded Sheet of the PLA Filaments

**Figure 3:**
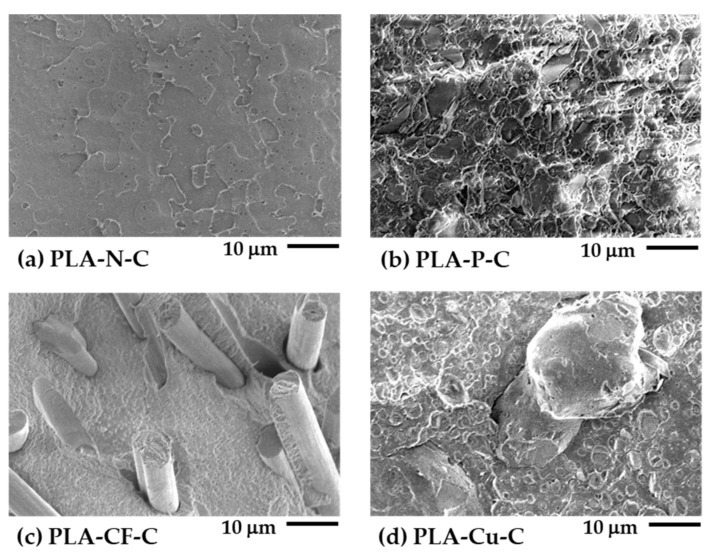
SEM images of cryogenic fractured surfaces of PLA filaments compression molded sheets: (**a**) PLA-N-C; (**b**) PLA-P-C; (**c**) PLA-CF-C; and (**d**) PLA-Cu-C.

Figure 3 depicts SEM images of cryogenic fractured surfaces of the compression molded sheet of the PLA filaments. The fractured surface of the PLA-N-C was smooth as indicated its brittleness as presented in Figure 3a. Figure 3b–d show morphology and characteristic of the additives on the PLA matrix. The particulate fillers in the PLA-P-C were good distribution and dispersion on the PLA matrix. The SEM image of the PLA-CF-C showed good adhesion of the CF on the matrix, which no CF fiber pulled out. Large copper particles and a kind of rubbery dispersed phase can be observed in the PLA-Cu-C. These SEM images could be encouraged the rheological properties, thermal properties, dynamic mechanical properties, and mechanical performance of the PLA composites 3D printings.

### 3.2. Rheological Properties

Rheological behaviors of the PLA and PLA composites were carried out to understand their flowability and viscoelastic properties under shearing since molten filaments were extruded passed through the hot nozzle. Rheological properties provide information of molecular entanglement and molecular relaxation, layer stability, and guideline of layer adhesion for FDM 3D printability [5,10,11]. 

**Figure 4:**
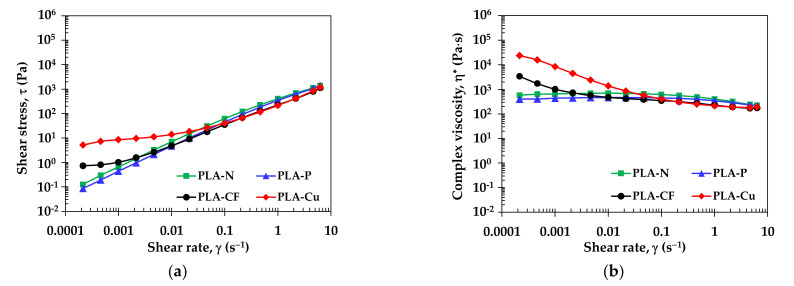
Flow curves of PLA filaments at 210 °C: (**a**) shear stress and (**b**) complex viscosity.

Flow curves of the compression molded sheets of PLA composites filaments at 210 °C are shown in Figure 4a for shear stress and Figure 4b for complex viscosity as a function of shear rate. At low shear rate, the shear stress and the complex viscosity of PLA-Cu and PLA-CF were higher than PLA-N and PLA-P. It was considered that CF and Cu inhibit movement of polymer molecule that raised their stress and viscosities. The high complex viscosity informs interaction between CF and Cu with the PLA matrix and increased elastic deformation and molecular entanglement of PLA-CF and PLA-Cu than PLA-N and PLA-P at the low shear rate. All filaments were pseudoplastic flow behavior. At high shear rate, the shear stress and the complex viscosity of all filaments revealed the shear thinning and the values were comparable regardless with the incorporation of additives. These indicated similarity of the PLA mainly matrix in the composite filaments [30]. Figure S1 presents the complex viscosity as a function of angular frequencies, which the viscosity characteristics of the PLA-N and PLA-P were also observed in Benwood et al. [5]. This research reported the complex viscosity of PLA filaments FDM 3D printing at various temperatures. From the rheological study, it was used to confirm the change of viscosity in the printing nozzle and informed the dimensional accuracy. Higher viscosity and proper control printing at 210 °C with bed temperature of 60 °C were optimized for high dimensional accuracy between the successive of voids between the layers in the 3D printings [5]. 

The power–law model as presented in Equation (2) is determined the flow behavior to identify 3D printing layer and dimensional stability [11,31], where *τ* is shear stress, $\dot{\gamma }$ is shear rate, K is consistency index, and *n* is power–law index, which were estimated by the power regression.

(2) $$\tau =Κ{\dot{\gamma }}^{n}$$

Table 3 summarizes viscosity, the power–law index and the consistency index of the filaments at 210 °C. The viscosity (η) values at two ranges of shear rates that related to flow behavior of the 3D printing filaments. The η values at 0.2 s^−1^ and 0.01 s^−1^ informed viscous flow of molten filament passing through the hot nozzle and during printed layer-by-layer, respectively [11]. It was surprisingly that the molten PLA composites have lower viscosities than the neat PLA. The viscosities of all molten filaments increased since the shear rate of the molten filaments decreased after extruded from the nozzle then layer deposition. The PLA-Cu has the highest viscosity along the layer deposition, which the layer might collapse from the high viscosity. The power–law index (n) of PLA-N and PLA-P were 0.91 and 0.95, which closed to the Newtonian flow (*n* = 1). On the contrary, PLA-CF and PLA-Cu have *n* < 1, which informed the shear thinning behavior. The consistency index (K) can be referred to the overall viscosity of the PLA composites filaments. The K values of the filaments were about 200–400 Pa·s. Nguyen, et al. [10] reported the window suggested for attaining good 3D printability by the shear rate about 190 s^−1^ to 3000 s^−1^ and the viscosity about 70 Pa·s to 500 Pa·s. In addition, the shear thinning with high viscosity could obtain the shape accuracy as reported in the literature [11]. Hence, the results confirmed that the PLA composites filaments would perform good printability. 

**Figure 5:**
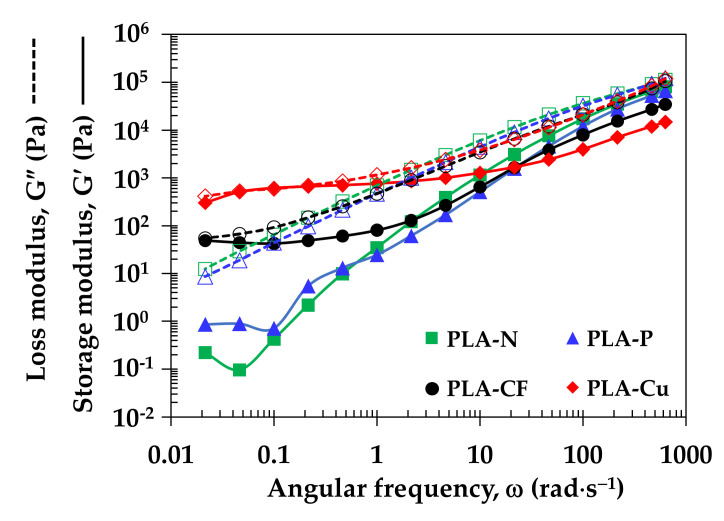
Storage modulus and loss modulus of PLA filaments.

Figure 5 displays storage and loss modulus under shearing of the PLA filaments. From the storage modulus (G’), the sudden drop of the G’ at low frequency of PLA-N and PLA-P indicated loss of elasticity, which implied molecular deterioration of the PLA matrix when processed at long time [22]. The storage moduli of the PLA composites were higher than PLA-N at the low frequency. It was attributed to interaction and good distribution of additives in the PLA matrix. In addition, the high values of G’ informed elastic deformation and molecular entanglement of PLA-CF and PLA-Cu at low frequency. It can be noted that the G’ and the loss modulus (G”) of PLA-Cu were equally at low frequency. It might inform the combination of solid–liquid transition behavior, which affected on molecular relaxation of the PLA-Cu filament [30]. From the loss modulus, all filaments were similarly in viscous behavior of the PLA matrix. Hence, the incorporation of the additives would stabilize molten PLA composites filaments during 3D printing [11]. 

### 3.3. Thermal Properties and Crystallization Behavior

**Figure 6:**
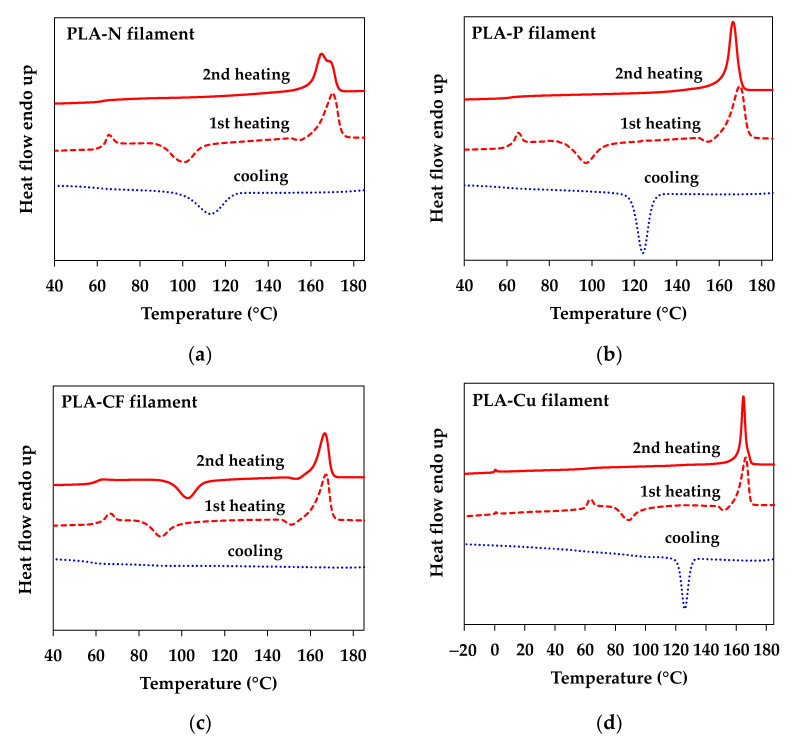
DSC thermograms of PLA filaments: (**a**) PLA-N; (**b**) PLA-P; (**c**) PLA-CF; and (**d**) PLA-Cu.

Figure 6 illustrates DSC thermograms of the PLA composites filaments from the first heating, the cooling and the second heating cycles to investigate the effect of additives on thermal properties of the PLA composites filaments. The thermograms from the first and the second heating cycles display glass transition temperature (T_g_), cold crystallization temperature (T_cc_), and melting temperature (T_m_) of PLA and the crystallization temperature (T_c_) at the cooling cycle. T_g_ of the PLA matrix around 60–64 °C can be clearly seen from the first heating while it was broad in the second heating as the PLA could be crystallized after removed thermal history and controlled the cooling rate [27]. An exothermic area at the heating cycles informed the T_cc_ that indicated slow crystallized and imperfect crystal of PLA matrix [23]. An endothermic peak around 165–170 °C of the first and the second heating cycles indicates T_m_ of the PLA matrix. The exothermic peaks of the cooling cycles are clearly seen in PLA-N, PLA-P, and PLA-Cu. The higher T_c_ and sharp intensities of the T_c_ and the T_m_ of the second heating peaks in the PLA-P and the PLA-Cu implied an improvement of PLA crystallization from the adding of particulate filler and Cu. On the other hand, the appearance of T_cc_ in the PLA-CF informed that CF retarded the crystallization of PLA. Nevertheless, semicrystalline PLA in the PLA-CF samples was crystallized, which small T_c_ of the PLA-CF can be seen when enlarging the cooling cycles as shown in Figure S2. It can be noted that PLA-Cu filament might be modified for improved filler distribution and flowability by rubbery additive, which can be observed the T_g_ around 0 °C in Figure 6d. This result could be confirmed the rubbery dispersed phase that observed in the morphology of the PLA-Cu-C in Figure 3d. The thermal properties of the PLA filaments were discussed with compression molded sheets and 3D printed samples. 

**Figure 7:**
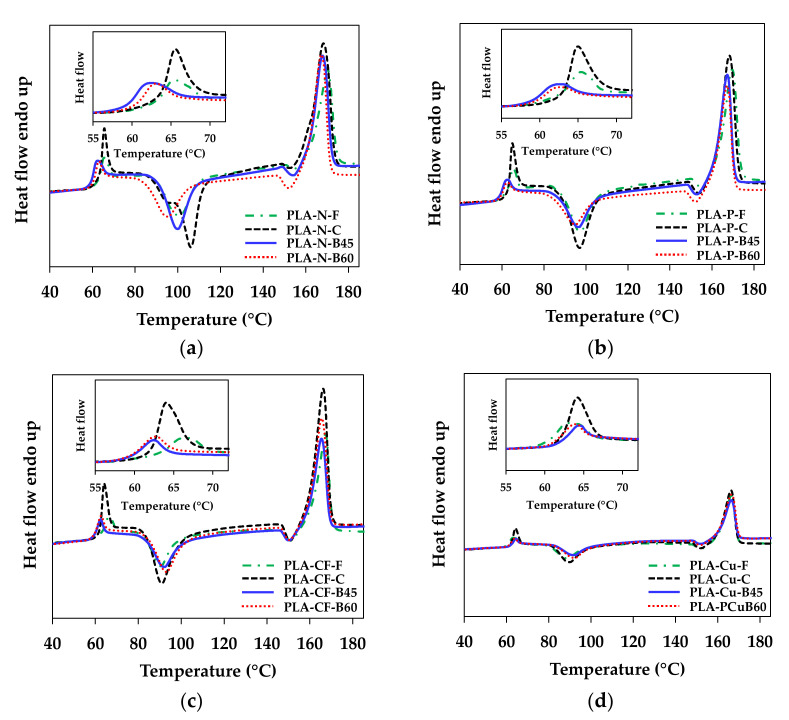
DSC thermograms of the first heating cycle of compression molded sheet and 3D printed samples: (**a**) PLA-N; (**b**) PLA-P; (**c**) PLA-CF; and (**d**) PLA-Cu.

Figure 7 depicts a comparison of the first heating DSC thermograms of the compression molded sheets, the 3D printed samples and their filaments to investigate the effect of thermal history from the additives and the processing on thermal characteristic of PLA. Table 4 summaries thermal properties of all samples. T_g_ values of the filaments were about 64 °C. Except PLA-Cu-F that was about 62 °C, which was due to the rubbery additive in this filament. The T_g_ values of the 3D printed samples were shifted to lower temperature as compared to the filaments and the compression molded sheets. It might be due to polymer mobility during the 3D printing process. However, the T_g_ of PLA-Cu 3D printings slightly shifted to higher temperature, which high amount of Cu might limit the mobility of polymer in the 3D printing. However, faster cooling rate in the compression molded reflected in higher amorphous region that indicated by larger intensities of the T_g_ and the T_cc_ area and lower in crystallinity as compared to the filaments and the 3D printed products. The incorporation of the additives and the increasing of the bed temperatures improved crystallization of the PLA matrix, which indicated by a decreasing of the T_cc_ and an increasing of crystallinity as tabulated in Table 4. The improvement of the PLA crystallization in the 3D printed products was more pronounced in PLA-N and PLA-P, especially when printed at bed 60 °C that would imply high stiffness of PLA-N-B60 and PLA-P-B60 samples. The T_m_ values of PLA in the compression molded sheets and the 3D printed samples were about 166–170 °C depend on PLA crystallization. The decreasing of T_m_ in the first heating cycle was due to crystal formation affected from the additives and the processing conditions. The crystallinity of the filaments and the 3D printing samples were higher than the compression molded sheets. It was attributed to the molecular orientation of PLA during extrusion process. 

According to the 3D printing process, there was much accumulation of heat when printed at higher bed temperature. Then, PLA might have smaller crystal formation from higher cooling rate when using higher bed temperature as compared to the lower one [18]. In the second heating after controlled cooling process, the T_g_ values were around 62 °C in all samples, which were assumed similarity of the PLA matrix in the neat PLA and the PLA composites filaments. The PLA-N samples presented two melting peaks while the other showed one T_m_ at 165–166 °C. It was corresponding to difference of crystal sizes in the pristine PLA-N during crystallization [32]. The T_m_ value in the second heating of each sample was unchanged, which implied no degradation of the PLA matrix after processing. The higher T_c_ of PLA-P and PLA-Cu than PLA-N informed that the particulate filler and the Cu acted as heterogeneous nucleating sites and enhanced the crystallization of the PLA matrix [5,27]. It can be noted that the T_c_ of the samples were hinted for layer solidification in the 3D printing process [22]. Higher T_c_ allows faster solidification that might restrict layer adhesion in the 3D printing. Hence, controlling in thermal and crystallization properties of PLA would design its printing quality and mechanical performance of the 3D printed products.

### 3.4. Dynamic Mechanical Properties

**Figure 8:**
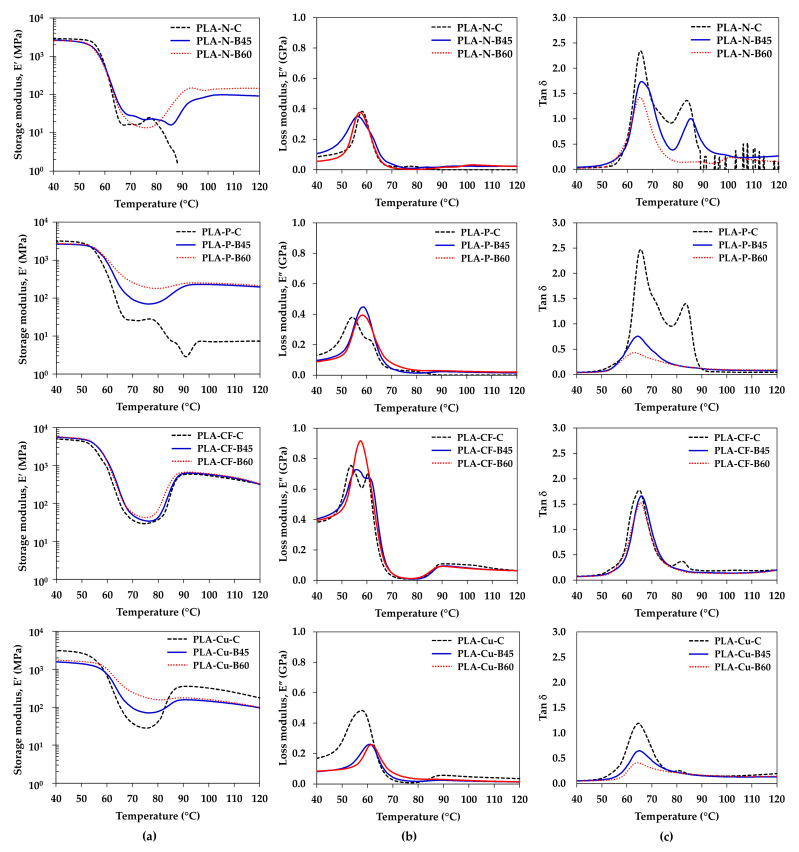
Storage modulus, loss modulus and Tan δ of compression molded sheets and 3D printed samples: (**a**) Storage modulus; (**b**) Loss modulus; and (**c**) Tan δ.

Dynamic mechanical properties inform the viscoelastic properties of polymers, which can be used to understand phase transition, molecular mobility and damping property of polymer, and compatibility as well as interfacial adhesion of polymer blends and composites [24,25,26,27,33]. Additionally, the dynamic mechanical properties can be implied the layer adhesion in the 3D printing products [19,34]. The effects of additives and processing conditions on the dynamic mechanical properties of the PLA composites compression molded sheets and the 3D printed samples are illustrated in Figure 8. The storage modulus (E’), loss modulus (E”) and Tan δ values of the samples are summarized in Table 5. Phase transitions of the materials according to molecular mobility from glassy region, glass transition region, rubbery region, and flow region can be indicated from changing of the E’ values at elevated temperatures. The E’ decreased when increasing temperature and sudden drop at the glass transition temperature toward the rubbery stage and increased viscous and chain mobility [25,26]. The compression molded of the PLA-N-C and the PLA-P-C lost their stiffness around 70 °C whereas the stiffness could be recovered in the other compression molding and the 3D printing samples from the cold crystallization [32]. The incorporation of the particulate filler and the Cu particle improved the crystallization of the PLA and rose the E’ at elevated temperature. Additionally, the E’ increased when increasing bed temperature from the crystallinity improvement. Storage moduli of the compression molded PLA-N-C, PLA-P-C and PLA-Cu-C were higher than their 3D printed samples because of good adhesion and the interaction between the additives and the PLA matrix. The PLA-CF-C, PLA-CF-B45 and PLA-CF-B60 showed the maximum E’ values, which were stiffer as compared to the other due to the reinforcement and stiffness of the CF. In addition, the anisotropic orientation of the CF along the extruded 3D printing yielded higher E’ than the PLA-CF-C one. Nevertheless, the Cu particles declined the E’ of the 3D printed PLA-Cu-B45 and PLA-Cu-B60, which might be due to the rubbery phase and limited layer adhesion. The E’ along the rubbery state can be informed thermal resistance of the polymers indicated by a shift of the E’ to higher temperature. From Figure 8a and the E’ values at 60 °C in Table 5, the 3D printed samples exhibited higher thermal resistance and harder than the compression molded sheets. Additionally, the PLA-N-C and PLA-P-C lost their stiffness at temperatures over 80 °C, which was probably attributed to highly amorphous state of the compression molded PLA-N-C and PLA-P-C. It can be confirmed that the incorporation of the additives and the 3D printing process enhanced the thermal resistance, the elasticity, and the stiffness of the 3D printed samples [11,19,33].

Figure 8b displayed E” curve of the PLA composites compression molded, and the 3D printed samples. The E” support information of viscous response of materials, energy dissipation and related to relaxation process [23,26]. The E” peaks of the 3D printed samples shift to higher temperatures. Higher E” values indicated more energy dissipation, which was attributed to an increment of internal friction in composites. E” of the PLA-P and PLA-CF composites were higher than the neat PLA. Akindoyo, et al. [23] reported that higher E” of the composites than neat PLA could be due to the higher segmentation of chains in the PLA matrix, which the reinforcing fillers might have hindered chain relaxations within the composite and larger number of energy dispersing spots formed by interfacial bonding. 

The damping property and the glass transition temperature of the samples were determined by Tan δ as presented in Figure 8c. All compression molded exhibited higher Tan δ, which the energy was more dissipate than the 3D printed samples. The damping property of the 3D printed samples improved when increasing the bed temperature that materials would have more potential to store energy than dissipation [19]. 

The storage modulus and the Tan δ values were used to investigate the degree of entanglement density (*N*), the adhesion factor (*A*), the effectiveness coefficient (*C*), and the reinforcing factor (*r*) of the fillers in the PLA composites samples. All parameters are calculated from Equations (3)–(6) [23,25,26,28,29,34] and the results tabulate in Table 5. 

The degree of entanglement density (*N*) can be calculated from the following [26,28]:

(3) $$N=\frac{E^{\prime }}{6RT}$$

where $E^{\prime }$ is the storage modulus at the rubbery stage (60 °C), *R* is the universal gas constant (8.314 J·mol^−1^·K^−1^), and *T* is the absolute temperature at the rubbery stage.

The adhesion factor (*A*) can be described interfacial interaction and interaction between filler and the adhesion with the polymer matrix by measuring from damping factor as presented in the Equation (4) [23,26]:

(4) $$A=\frac{1}{\left(1-V_{f}\right)}\left(\frac{\tan\delta_{c}}{\tan\delta_{p}}\right)-1$$

where $V_{f}$ is volume fraction of the filler, and tan $\delta_{c}$ and tan $\delta_{p}$ is the maximum value of tan δ peak of the composite and the neat polymer, respectively. 

The effectiveness coefficient (*C*) can be calculated from the ratio of the storage modulus of the glassy stage ($E_{g}^{\prime }$) and the rubbery stage ($E_{r}^{\prime }$) between the composite and the neat polymer as presented in the following equation [23,25,26,29,34]:

(5) $$C=\frac{\left(\frac{E_{g}^{\prime }}{E_{r}^{\prime }}\right)\mathrm{composite}}{\left(\frac{E_{g}^{\prime }}{E_{r}^{\prime }}\right)\mathrm{neat}\;\mathrm{polymer}}$$

The reinforcement efficiency factor (*r*) of the composite was investigated according to the rule of mixture from the Einstein equation [26,28].

(6) $$E_{c}^{\prime }=E_{m}^{\prime }\left(1+rV_{f}\right)$$

where $E_{c}^{\prime }$ and $E_{m}^{\prime }$ are the storage modulus of the composite and the polymer matrix, respectively and $V_{f}$ is volume fraction of the filler. 

The degree of entanglement density would inform the effect of additives and the processing condition on molecular entangle in the neat polymer and the composites. The degree of entanglement density of the neat polymer was comparable in both compression molding and 3D printing processes. Higher content of the CF and the Cu obtained higher entanglement in the compression molded PLA-CF-C and PLA-Cu-C as compared to PLA-N-C and PLA-P-C. This information was correlated with the rheological properties. The entanglement of the PLA-N was comparable in the compression molded and the 3D printed specimen. On the other hand, the molecular entanglement of the PLA composites increased in the 3D printed samples and increased when increasing the bed temperature. It was considered that molecular mobility was higher during layer deposition in the 3D printing as compared to the fast cooling in the compression molding process. Hence, the higher degree of entanglement density can be informed better layer adhesion in the 3D printing process. Nevertheless, the mechanical performance of the 3D printing might be limited by the anisotropic characteristic of the filler in the composite and the 3D printing direction [11]. 

The adhesion factor was calculated in the compression molded sampled to inform the adhesion and the interaction between the fillers and the PLA matrix. The lower value of the adhesion factor in the PLA-CF-C indicated the higher degree of the interfacial adhesion and interfacial interaction between the CF and the PLA matrix [23,26]. The level of the adhesion factor of the particulate filler in the PLA-P-C was in the middle. The PLA-Cu-C has the high value of the adhesion factor, which the Cu particle was less interfacial adhesion with the PLA matrix as compared to the other. The effectiveness of the fillers in the composites was investigated by the effectiveness coefficient from the ratio between the E’ of the glassy region (at 40 °C) and the rubbery region (at 60 °C). Herein, the ratio was comparison with the neat polymer of each processing condition. Higher C indicates the lower effectiveness of the filler. From the results, the high content and large particle size of the Cu particle restricted molecular mobility in the compression molded PLA-Cu-C but obtained higher effectiveness due to the molecular entanglement as reported in the rheological properties and the degree of entanglement density. While the effectiveness of the particulate filler in the PLA-P-C and the CF in the PLA-CF-C was less efficient than the Cu due to the distribution of the fillers in the PLA matrix. On the contrary, the effectiveness of the fillers was increased in the 3D printing samples and increased with increasing the bed temperature, which was attributed to higher degree of entanglement density [26,28]. 

Additionally, the reinforcing efficiency factor was determined to elucidate the potential of the fillers and the effect of the processing conditions on mechanical performance of the composites. According to the characteristic and properties of the filler, the CF exhibited the highest reinforcing effect in the composites from its stiffness and good distribution and interaction with the polymer matrix [23,26,28]. Moreover, the orientation of the CF along the layer deposition in the 3D printer obtain better reinforcing effect of the 3D printed PLA-CF. The good distribution of the particulate filler would support its reinforcing efficiency in the PLA-P composites. However, less adhesion and large particle size of the Cu exhibited low reinforcing efficiency in the PLA-Cu composites. It was noted that the reinforcing efficiency of the fillers in the 3D printed samples increased when increasing the bed temperature due to the increment of molecular entanglement and interaction of fillers and the PLA matrix [23,26,28]. 

**Figure 9:**
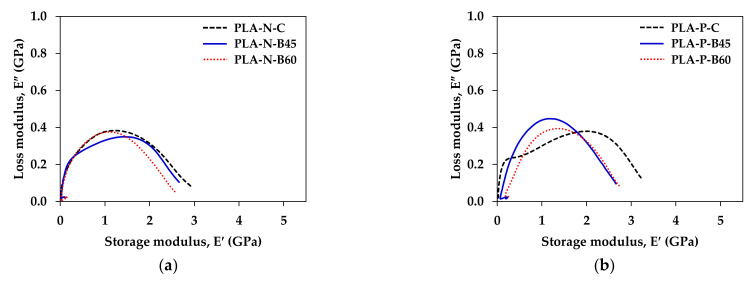

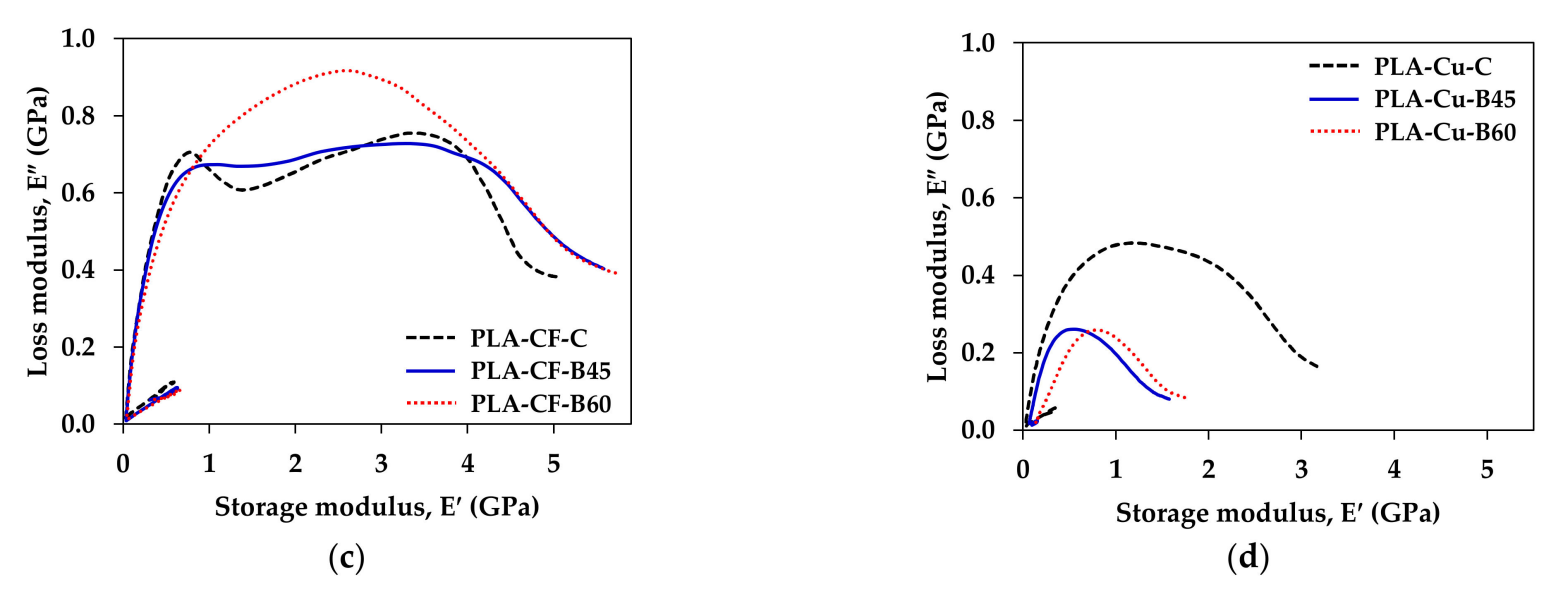
Cole–Cole plot of compression molded sheet and 3D printed samples: (**a**) PLA-N; (**b**) PLA-P; (**c**) PLA-CF; and (**d**) PLA-Cu.

Furthermore, the Cole–Cole analysis is obtained by plotting between the loss modulus and the storage modulus to describe homogeneity and change in the structural properties of the material system [25,26,33]. Figure 9 displays the Cole–Cole plot of the compression molded and the 3D printed PLA composites. The plot exhibited imperfect semicircle arc curves of all PLA composites, which informed the heterogeneity in the neat PLA and the composites and indicating good interfacial bonding in the composites [24]. The imperfection was improved in the 3D printing process due to the increment of the interfacial interaction and adhesion [25,33]. Devi et al. [25] and Jyoti et al. [26] reported that the shape of the Cole–Cole plot points relatively good adhesion between the matrix with glass fiber and MWCNT, respectively.

### 3.5. Mechanical Properties

**Figure 10:**
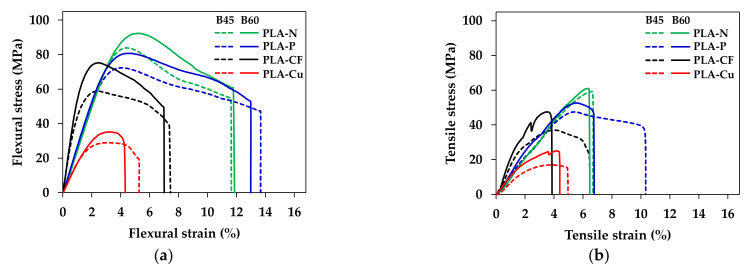
Typical stress–strain curves of 3D printed samples: (**a**) flexural test and (**b**) tensile test.

**Figure 11:**
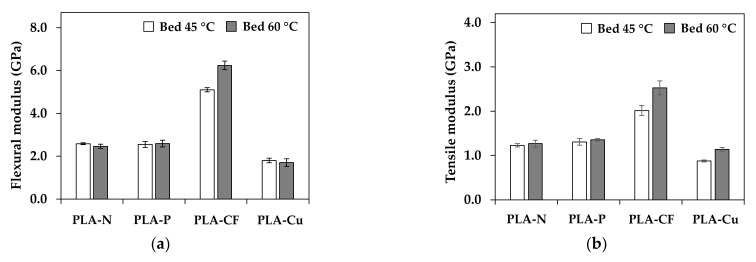

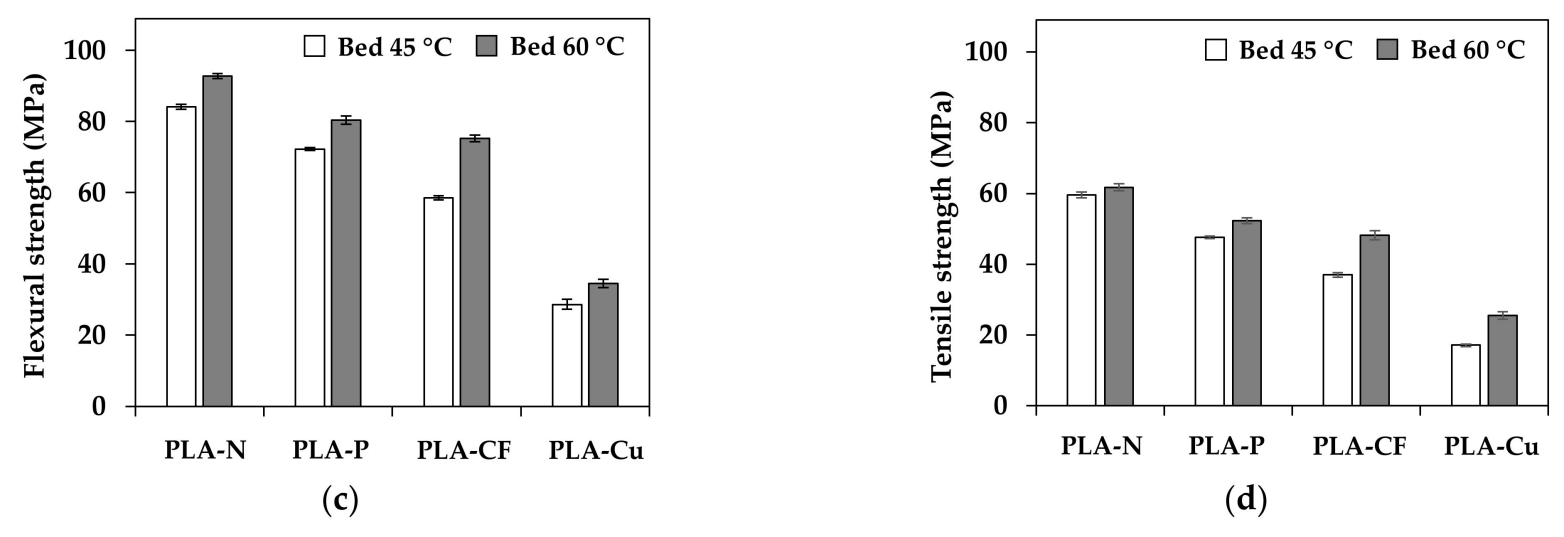
Flexural and tensile properties of 3D printed samples: (**a**) flexural modulus; (**b**) flexural strength; (**c**) tensile modulus; and (**d**) tensile strength.

Static mechanical properties were carried out by flexural and tensile testing. Figure 10 shows typical stress-strain curves of the 3D printed samples from bed temperatures of 45 °C (B45) and 60 °C (B60). The stress increased whereas ductility of the composites decreased with increasing the bed temperature as presented in Figure 10. Flexural and tensile properties are depicted in Figure 11. 

The 3D printed at B60 obtained better stiffness and resistance of bending and tensile loading indicated by higher modulus and strength, which were due to the increment of the reinforcing efficiency factor at higher bed temperature. On the contrary, the ductility and toughness of the 3D printed promoted in the B45 samples. In the viewpoint of the incorporation of additives and layer adhesion in 3D printing, flexural and tensile modulus of the PLA-P and the PLA-CF increased by the rigidity of particulate filler and carbon fiber as presented in Figure 11a,b. In addition, the CF orientation, and its content significantly improved modulus of elasticity [20]. However, mechanical properties of the PLA-Cu were low due to less continuous of PLA matrix to withstand flexural and tensile loading from the restriction of high content of the Cu particle and the low reinforcing efficiency factor. Flexural and tensile strength of the composites were lower than the neat PLA, as shown in Figure 11c,d. It was due to the interaction between the fillers and the matrix, which limited load transferring [23] resulting in the declination of the strengths as compared to the neat PLA. It is noteworthy that the interaction and the adhesion of the fillers and the matrix yield higher mechanical properties. Therefore, the design of the 3D printing would support mechanical performance although the layer adhesion in the 3D printing might be restrict. 

### 3.6. Morphology of the 3D Printed PLA Composites

**Figure 12:**
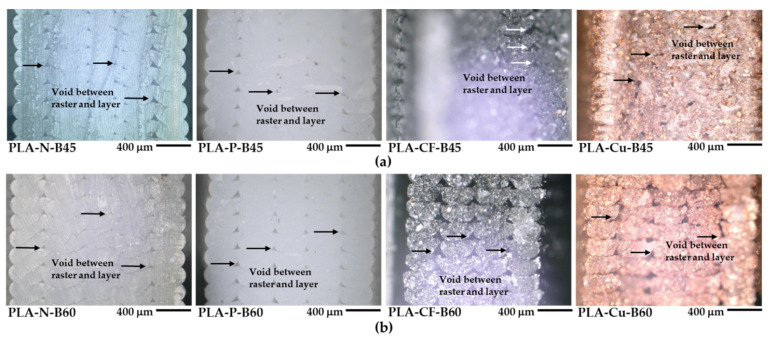
Optical micrographs of 3D printed samples: (**a**) at bed 45 °C and (**b**) at bed 60 °C.

Figure 12a,b display optical micrographs of the 3D printed samples at B45 and B60, respectively. Triangle voids between raster and printed layer can be seen in all 3D printed samples that indicated incomplete layer adhesion in the 3D printing process [5]. The void areas became larger in the PLA composites as compared to the neat PLA, regardless on the bed temperature. Generally, smaller void areas indicated good layer adhesion of the 3D printed samples [5,6]. Thus, fewer voids in the PLA-N implied better layer adhesion than the PLA composites 3D printings. It was considered from the effect of higher crystallization temperatures from heterogeneous additives induced faster layer solidification of molten PLA composites and may restrict layer adhesion in the PLA composites 3D printing [22]. Nevertheless, the voids of the PLA-P were small and probably have good layer adhesion comparable to the PLA-N. It was considered that the PLA-P has high degree of entangle density and has similar flow behavior as compared to the PLA-N, while high content of the Cu particles and the flow with the rubbery phase interfered the fractured surface in the PLA-Cu-B45 and B60, which were covered existed voids. On the contrary, although the CF retarded PLA crystallization, it was the heterogeneity induced the PLA solidification resulting of large voids appeared in the PLA-CF 3D printing structure. 

Figure S3 shows densities of the PLA filaments and their compression molded and the 3D printed samples. The densities of the samples slightly changed from the filament fabricated to the compression molded sheets and the 3D printed samples. The incorporation of the additives increased the densities of the PLA composites and improved crystallinity, dynamic mechanical, flexural, and tensile properties of the polymer composites. Furthermore, the porosity inside the 3D printed samples could be implied from the density measurement [5]. The density values were unchanged in the PLA-N-B45 and the PLA-N-B60, which they were comparable in the porosity from the setting bed temperatures. The densities of the 3D printed samples slightly increased when increasing the bed temperature in the PLA-P and the PLA-CF 3D printing. It might be informed small porosity or fewer voids in the PLA-P-B60 and the PLA-CF-B60 as compared to the 3D printing at bed 45 °C. On the other hand, the PLA-Cu has lower porosity when printing at bed 45 °C. Hence, the reduction of the porosity or voids implied better layer in the 3D printings that enhanced the dimensional accuracy and the mechanical performance of the PLA composites 3D printings [5]. 

**Figure 13:**
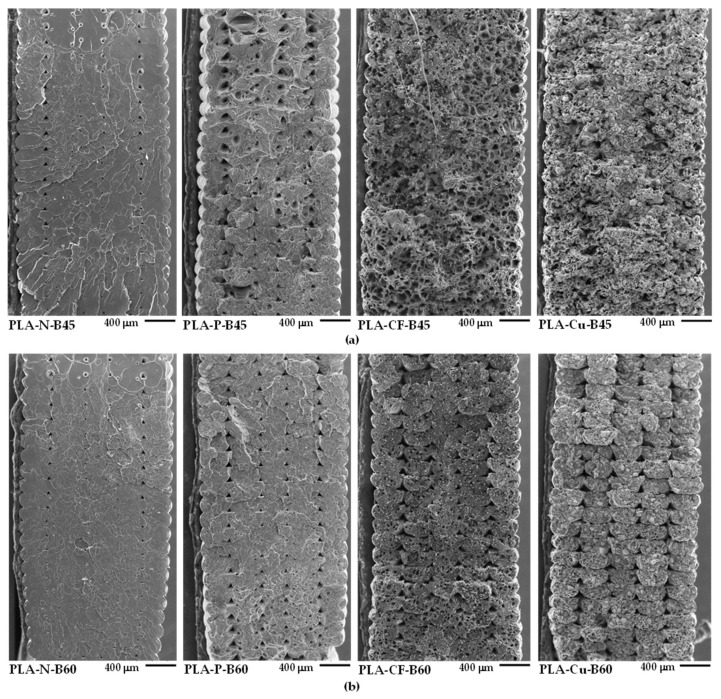
SEM images of tensile fractured surfaces of 3D printed samples: (**a**) at bed 45 °C and (**b**) at bed 60 °C.

The effects of additives and the bed temperatures on the layer formation and the fracture behavior of the PLA composites 3D printings were observed from the tensile fractured surface. Figure 13a,b reveal the SEM images at low magnification of the 3D printed samples at B45 and B60, respectively, which the bottom of the picture closed to the printed bed. Skin-core morphology was observed in the fractured surface the PLA-N-B45 and the PLA-N-B60, in which whitening appeared at the skin of the PLA-N samples. On the other hand, the printed bed significantly affected on the fracture behavior of the PLA composites filament. The SEM images revealed ductile and brittle fracture surfaces exhibited in B45 and B60, respectively. It was considered that molecular relaxation of PLA composites at B45 was higher than B60, which indicated by intensities of the Tan δ [23]. Then the relaxation allowed molecular movement during deformation resulting higher ductility of the PLA composites 3D printing at B45. While the composites printed at B60 were brittle, which reflected from the stiffness of the composites. From the SEM images, shrinkage slightly occurred at the bottom layers of the PLA-N-B60 and the PLA-P-B60. Therefore, it should be careful when printed on the bed temperatures higher than T_g_ of the PLA to increase layer adhesion and maintain the dimensional stability of the PLA composites 3D printing. 

**Figure 14:**
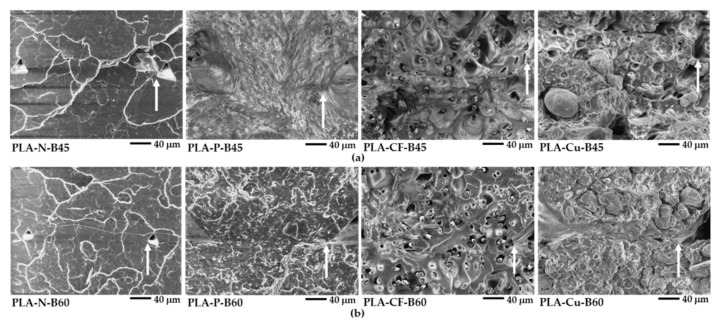
SEM images of tensile fractured surfaces of 3D printed samples at high magnification (×500): (**a**) at bed 45 °C and (**b**) at bed 60 °C.

**Figure 15:**
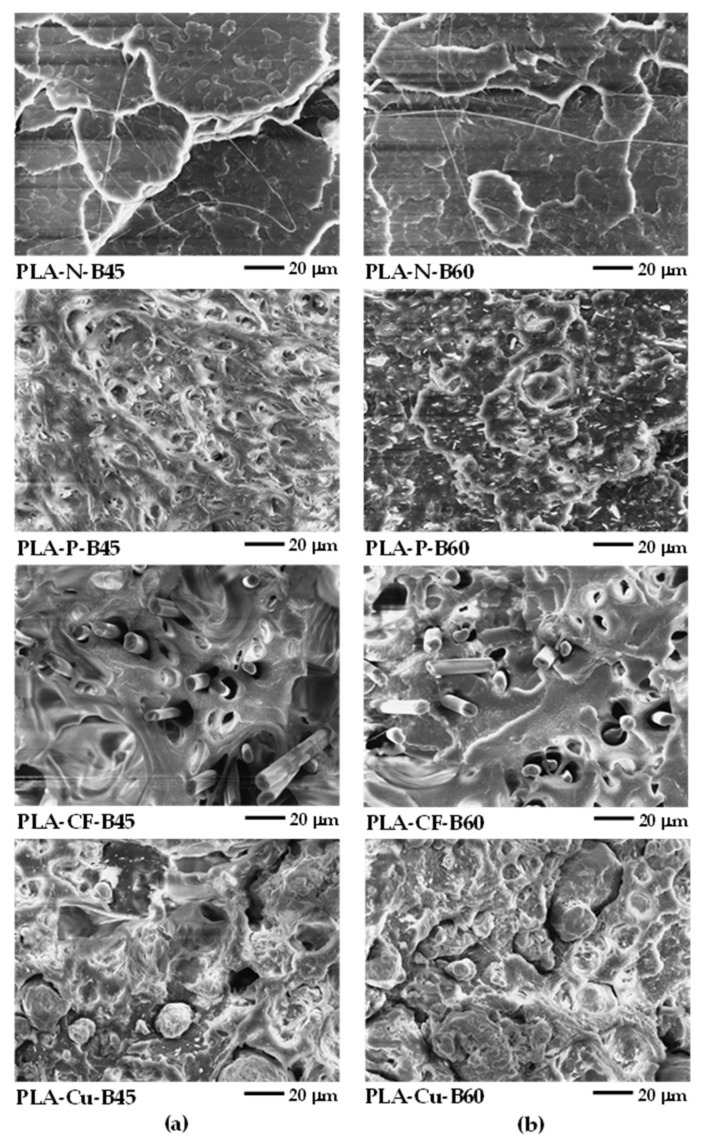
SEM images of tensile fractured surfaces of 3D printed samples at high magnification (×1000): (**a**) at bed 45 °C and (**b**) at bed 60 °C.

Figure 14 and Figure 15 present SEM images at high magnification of the tensile fractured surfaces. The layer adhesion of the 3D printed sample was observed from the adjacent between the printed layers as shown in Figure 14. The boundary of the adjacent between printed layers can be observed from the corner of the triangle voids as indicated by the arrows, which presented the layer adhesion of the 3D printing. The neat PLA has fewer voids and no boundary of the adjacent layers, which implied superior layer adhesion of the PLA-N-B45 and the PLA-N-B60. The boundary informed the incomplete adhesion of the printed layer in the PLA composites. It was due to rapid solidification induced from the reinforced additives indicating less adhesion than the neat PLA. Although the layer adhesion in the PLA composites 3D printing was low, the molecular entanglement, the interaction of the filler and the matrix, and the reinforcing efficiency of the fillers obtained high mechanical properties that were supported their applications as compared to the neat PLA. 

From Figure 15, both PLA-N-B45 and PLA-N-B60 showed smooth of brittle fracture surface. Nevertheless, the elongated fibrils supported toughening and resistance to fracture in the 3D printing of the PLA-N [35]. Large deformation of the samples printed at B45 verified their ductility as depicted in Figure 15a. The elongated deformation and debonding between the additives and the PLA matrix in the PLA-P-B45 and the PLA-CF-B45 confirmed the ductility and toughening of these samples. The interfacial adhesion and the rigidity of the particulate filler and the carbon fiber improved modulus of the PLA-P-B60 and the PLA-CF-B60 even the existing of the voids. Moreover, the orientation of the carbon fiber along the 3D printing insisted the reinforcing ability in this printing direction. The SEM images of the PLA-Cu-B45 and B60 showed the rubbery dispersed phase and large copper particles that influenced on the printing ability and their layer adhesion. However, high contents of the Cu particles could support the dimensional stability and provided copper-like appearance whereas affected in low adhesion due to less PLA matrix. Thus, mechanical properties of these PLA-Cu 3D printing were poor as compared to the compression molded sheet of the PLA-Cu-C. It can be noted that the ductility of the PLA composites 3D printing improved at the lower bed temperatures owing to higher molecular relaxation [19,23]. The higher bed temperature enhanced modulus and strength of the PLA composites 3D printing. Hence, the combination of the materials’ characteristics, i.e., rheological behavior and incorporation of additives and the 3D printing conditions such as printing directions, printing bed temperatures, infill, layer thickness, and so on to obtain superior mechanical properties of the 3D printing products.

## 4. Conclusions

Rheological behaviors and dynamic mechanical properties are powerful to understand the layer adhesion and the effect of additives and the bed temperatures on the properties of the PLA composites 3D printing. The Newtonian and the shear thinning flows with the moderate viscosity yielded layer stability of the molten polymer, the dimension stability, and printability of the 3D printing products. The dynamic mechanical properties informed the viscoelastic properties, the interfacial adhesion, and the interpretation of the layer adhesion in the 3D printing. The incorporation of the additives influenced on the molecular relaxation, the molecular entanglement, and the interfacial adhesion in the PLA composites. The degree of entanglement density increased when incorporated with the fillers and increasing the bed temperature. The particulate filler and the carbon fiber exhibited reinforcing efficiency and enhanced mechanical performance in these PLA composites 3D printings. However, high content of the copper particle limited the layer adhesion resulting the declination of the mechanical properties of the PLA-Cu 3D printings. The increment of the molecular relaxation in the lower bed temperature at 45 °C obtained high ductility and good toughness. On the contrary, the enhancing of the crystallinity, the interfacial interaction and the degree of entanglement density promoted stiffness and improved flexural and tensile properties when printed at higher bed temperature of 60 °C. The morphology of the PLA composites indicated incomplete and restrict of layer adhesion when incorporated with the fillers and the fiber additives. Nevertheless, the reinforcing effect, the good interfacial adhesion of the fillers with the PLA matrix and the molecular relaxation could be optimized by the incorporation of the additives and the suitable 3D printing conditions for obtaining superior mechanical performance of the PLA composites 3D printing. Nevertheless, the layer adhesion might be limited from the anisotropic characteristic of the composites and the layer-by-layer in the 3D printing. Therefore, the information in this research provides a guideline for develop new filament feed stocks, i.e., with the particulate filler, reinforcing fiber and metal powder, and processing optimization for high quality polymer composites 3D printing. 

## Figures and Tables

**Table 1:** Designation and composition of PLA filaments.

Designation	Polymer	Additive	Residual ^1^ (%)	T_d5%_ ^1^ (°C)	T_dpeak_ ^1^ (°C)
PLA-N	PLA	-	0.0	317.6	353.0
PLA-P	PLA	Particulate filler	5.2	317.3	352.5
PLA-CF	PLA	Carbon fiber	14.0	305.4	348.9
PLA-Cu	PLA	Copper particle	66.4	311.4	343.3

^1^ Residual content at 500 °C and decomposition temperature (T_d5%_ and T_dpeak_) from TGA.

**Table 2:** Conditions of FDM 3D printing.

Parameter	FDM 3D Printing Condition
Nozzle temperature	210 °C
Bed temperature	45 °C and 60 °C
Printing speed	25 mm/s
Layer height	0.2 mm
Shell thickness	2 layers
Raster angle	0°
Infill type	Rectilinear
Infill density	100%

**Table 3:** Complex viscosity and power–law index of PLA filaments at 210 °C.

Sample	$\eta \;\mathrm{at}\;\dot{\gamma }$ 0.2 s^−1^ (Pa·s)	$\eta \;\mathrm{at}\;\dot{\gamma }$ 0.1 s^−1^ (Pa·s)	K (Pa·s)	*n*	Power Regression (R^2^)
PLA-N	555.5	694.6	381.2	0.91	0.99
PLA-P	426.2	464.7	324.2	0.95	0.99
PLA-CF	313.7	474.5	215.2	0.76	0.99
PLA-Cu	305.3	1377.5	222.1	0.52	0.94

**Table 4:** Thermal properties and crystallinity of filaments, compression molded sheets, and 3D printed samples.

Sample	DSC First Heating Cycle				DSC Cooling Cycle		DSC Second Heating Cycle			
	T_g1_ (°C)	T_cc1_ (°C)	T_m1_ (°C)	X_c1_ (%)	T_c_ (°C)	ΔH_c_ (J/g)	T_g2_ (°C)	T_cc2_ (°C)	T_m2_ (°C)	X_c2_ (%)
PLA-N-F	64.1	101.0	170.1	8.5	112.6	37.8	60.3	-	165.0, 168.8	35.8
PLA-N-C	64.1	106.1	168.3	3.7	108.2	30.6	61.7	99.5	163.8, 168.6	34.2
PLA-N-B45	60.6	99.9	167.9	5.3	111.1	32.7	62.0	-	164.3, 168.5	35.5
PLA-N-B60	61.8	95.2	167.3	11.6	112.4	32.6	61.9	-	164.1, 168.3	34.6
PLA-P-F	64.0	97.3	169.6	7.7	124.0	36.9	62.4	-	166.4	42.5
PLA-P-C	63.4	97.0	168.3	5.8	124.2	37.7	61.8	-	166.5	42.7
PLA-P-B45	60.5	96.4	167.3	8.3	123.7	36.7	63.2	-	166.0	41.8
PLA-P-B60	60.9	95.0	167.2	10.5	123.7	36.7	62.5	-	165.7	42.2
PLA-CF-F	64.6	90.4	167.2	11.8	91.3	0.8	60.6	102.9	166.7	9.2
PLA-CF-C	62.7	90.9	166.2	8.4	90.2	2.0	60.2	99.4	166.3	9.7
PLA-CF-B45	60.9	92.1	165.4	9.2	91.5	1.5	60.9	103.0	166.3	8.8
PLA-CF-B60	61.1	93.1	165.5	9.0	92.1	1.0	60.4	103.1	166.2	8.3
PLA-Cu-F	61.8	89.0	166.4	11.2	126.0	10.7	61.9	-	164.8	30.4
PLA-Cu-C	62.8	89.7	166.3	9.1	126.9	11.3	61.8	-	164.5	31.1
PLA-Cu-B45	63.4	92.1	166.4	11.6	126.6	11.3	62.0	-	164.7	30.1
PLA-Cu-B60	62.8	92.2	166.4	11.1	126.3	11.0	62.1	-	164.7	30.9

**Table 5:** Dynamic mechanical properties of compression molded sheets and 3D printed samples.

Sample	E’ (GPa) at 40 °C	E’ (GPa) at 60 °C	E” (MPa) ^1^	T_g_, Tan δ ^2^ (°C)	Tan δ ^2^	*N* (mol/m^3^)	*A*	*C*	*r*
PLA-N-C	2.91	0.48	0.38	65.6	2.33	2.89 × 10^4^			
PLA-N-B45	2.65	0.46	0.35	65.6	1.74	2.76 × 10^4^			
PLA-N-B60	2.56	0.38	0.37	65.0	1.42	2.31 × 10^4^			
PLA-P-C	3.21	0.43	0.38	65.8	2.47	2.58 × 10^4^	0.12	1.23	2.01
PLA-P-B45	2.66	1.07	0.45	64.4	0.76	6.41 × 10^4^		0.43	0.02
PLA-P-B60	2.72	1.24	0.39	63.2	0.43	7.48 × 10^4^		0.33	1.23
PLA-CF-C	5.00	0.77	0.75	64.8	1.77	4.63 × 10^4^	−0.12	1.07	5.13
PLA-CF-B45	5.56	1.32	0.73	65.9	1.66	7.93 × 10^4^		0.73	7.82
PLA-CF-B60	5.68	1.38	0.92	65.6	1.52	8.29 × 10^4^		0.61	8.72
PLA-Cu-C	3.13	0.62	0.48	64.7	1.19	3.73 × 10^4^	0.52	0.83	0.12
PLA-Cu-B45	1.57	0.74	0.26	65.2	0.65	4.45 × 10^4^		0.37	−0.62
PLA-Cu-B60	1.74	0.99	0.26	64.5	0.41	5.96 × 10^4^		0.26	−0.49

^1^ Peak of loss modulus. ^2^ Peak of Tan δ.

## Data Availability

The data presented in this study are available on request from the corresponding author.

## Supplementary Materials

The following supporting information can be downloaded at: https://www.mdpi.com/article/10.3390/polym14132721/s1. Figure S1. Complex viscosity as a function of angular frequency of PLA filaments. Figure S2. DSC thermograms of the enlarging cooling cycles of PLA-CF samples: (a) PLA-CF-F; (b) PLA-CF-C; (c) PLA-CF-B45; (d) PLA-CF-B60. Figure S3. Density of samples: (a) PLA-N; (b) PLA-P; (c) PLA-CF; (d) PLA-Cu.
