# Supplementary material for: Rheological Behavior and Dynamic Mechanical Properties for Interpretation of Layer Adhesion in FDM 3D Printing
Source: Polymers (Basel). 2022 Jul 3;14(13):2721. doi: 10.3390/polym14132721 (PMC9268954; doi:10.3390/polym14132721)
Supplement: Supplementary file 1 [file polymers-14-02721-s001.zip › polymers-1780831-supplementary.pdf]

polymers

Article

Rheological Behavior and Dynamic Mechanical Properties for Interpretation of Layer Adhesion in FDM 3D Printing

Supaphorn Thumsorn [1,*], Wattanachai Prasong [2], Takashi Kurose [1,3], Akira Ishigami [1,4], Yutaka Kobayashi [1] and Hiroshi Ito [1,4,*]

1 Research Center for GREEN Materials and Advanced Processing, Yamagata University, 4-3-16 Jonan, Yonezawa, Yamagata 992-8510, Japan; kurose.takashi@sist.ac.jp (T.K.); akira.ishigami@yz.yamagata-u.ac.jp (A.I.); kobayashi.y@yz.yamagata-u.ac.jp (Y.K.)
2 Department of Industrial Engineering, Faculty of Engineering, Pathumwan Institute of Technology, 833 Rama I Road, Wangmai, Pathumwan, Bangkok 10330, Thailand; wattanachai@pit.ac.th
3 Department of Mechanical Engineering, Faculty of Science and Technology, Shizuoka Institute of Science and Technology, 2200-2 Toyosawa, Fukuroi, Shizuoka 437-8555, Japan
4 Graduate School of Organic Materials Science, Yamagata University, 4-3-16 Jonan, Yonezawa, Yamagata 992-8510, Japan
* Correspondence: thumsorn@yz.yamagata-u.ac.jp (S.T.); ihiroshi@yz.yamagata-u.ac.jp (H.I.); Tel: +81-(23)-8263081 (H.I.)

Citation: Thumsorn, S.; Prasong, W.; Kurose, T.; Ishigami, A.; Kobayashi, Y.; Ito, H. Rheological Behavior and Dynamic Mechanical Properties for Interpretation of Layer Adhesion in FDM 3D Printing. *Polymers* **2022**, *14*, 2721. https://doi.org/10.3390/polym14132721

Academic Editors: Andrea Sorrentino

Received: 5 June 2022
Accepted: 1 July 2022
Published: 3 July 2022

Publisher's Note: MDPI stays neutral with regard to jurisdictional claims in published maps and institutional affiliations.

Copyright: © 2022 by the authors. Licensee MDPI, Basel, Switzerland. This article is an open access article distributed under the terms and conditions of the Creative Commons Attribution (CC BY) license (https://creativecommons.org/licenses/by/4.0/).

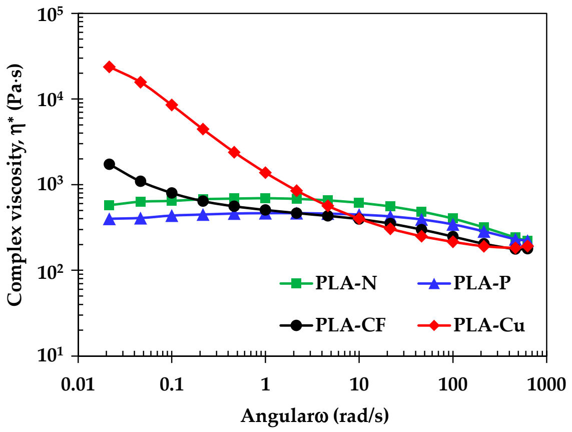

Figure S1. Complex viscosity as a function of angular frequency of PLA filaments.

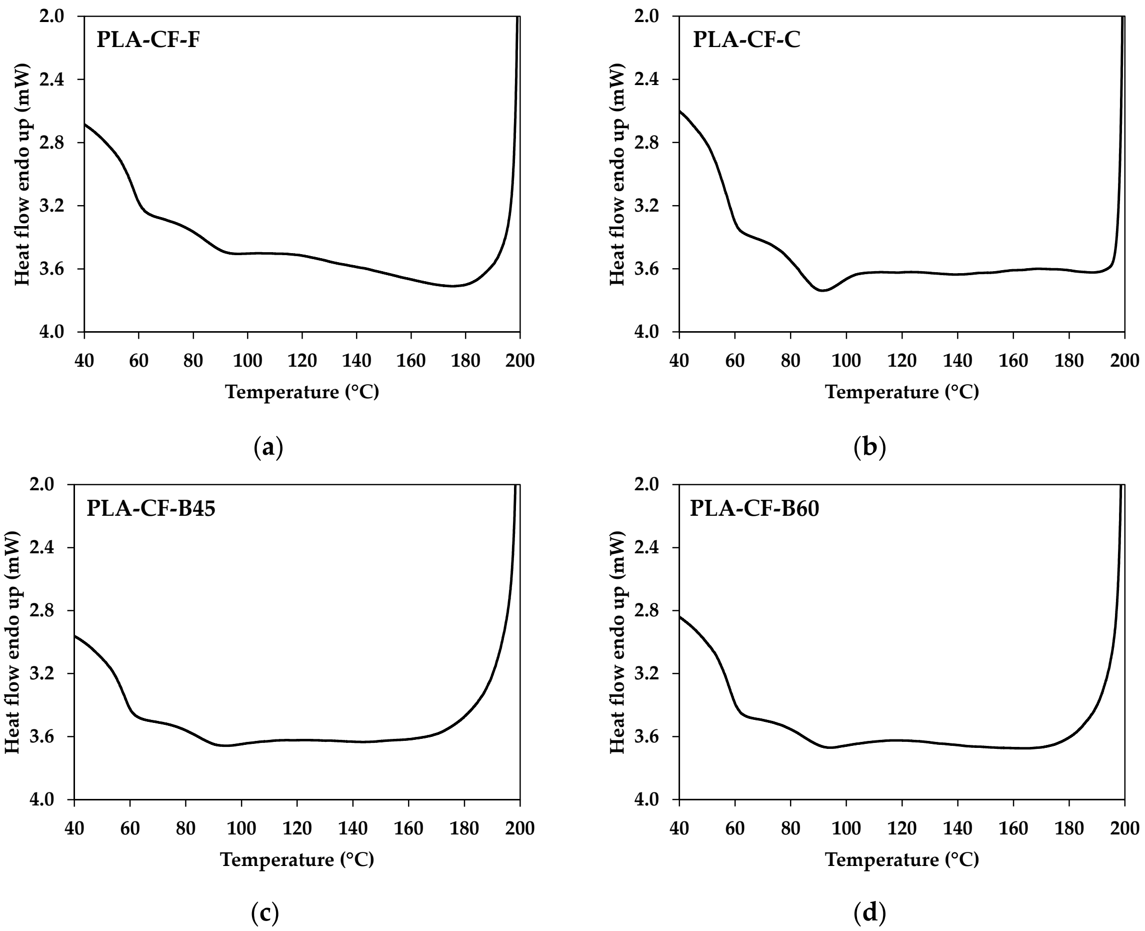

Figure S2. DSC thermograms of the enlarging cooling cycles of PLA-CF samples: (**a**) PLA-CF-F; (**b**) PLA-CF-C; (**c**) PLA-CF-B45; (**d**) PLA-CF-B60.

Density Measurement

Densities of samples were carried out by a gas displacement pycnometer (Micromeritics AccuPyc II 1345, Micromeritics Instrument Corporation, Norcross, GA, USA). The sample about 0.5–1.0 g was put in the sample cap. The densities of the samples were measured at room temperature under helium atmosphere.

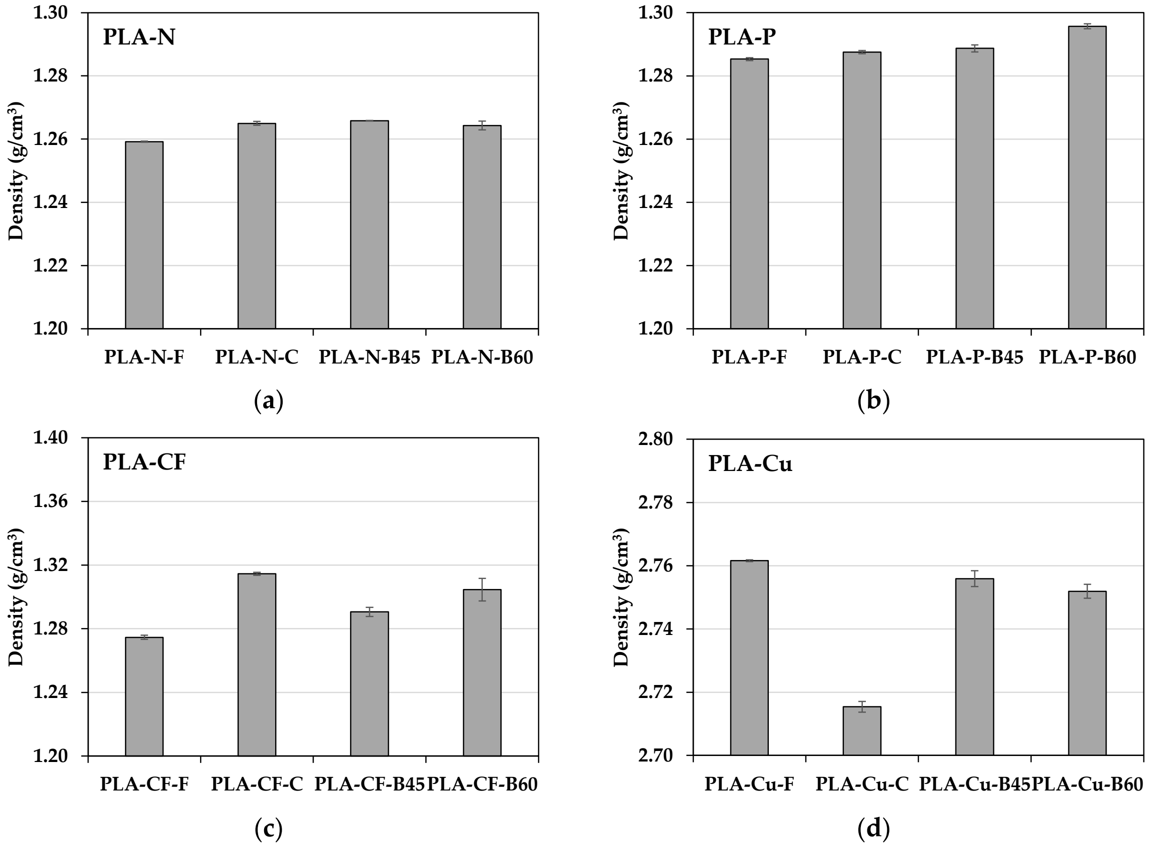

Figure S3. Density of samples: (**a**) PLA-N; (**b**) PLA-P; (**c**) PLA-CF; (**d**) PLA-Cu.
